# European hospitals as source of multidrug-resistant bacteria: analysis of travellers screened in Finland after hospitalization abroad

**DOI:** 10.1093/jtm/taac022

**Published:** 2022-03-02

**Authors:** Mikael Kajova, Tamim Khawaja, Anu Kantele

**Affiliations:** Department of Infectious Diseases, Inflammation Center, University of Helsinki and Helsinki University Hospital, Helsinki, Finland; Meilahti Infectious Diseases and Vaccine Research Center, MeiVac, University of Helsinki and Helsinki University Hospital, Helsinki, Finland; Multidisciplinary Center of Excellence in Antimicrobial Resistance Research, University of Helsinki, Helsinki, Finland; Department of Infectious Diseases, Inflammation Center, University of Helsinki and Helsinki University Hospital, Helsinki, Finland; Meilahti Infectious Diseases and Vaccine Research Center, MeiVac, University of Helsinki and Helsinki University Hospital, Helsinki, Finland; Multidisciplinary Center of Excellence in Antimicrobial Resistance Research, University of Helsinki, Helsinki, Finland; Department of Infectious Diseases, Inflammation Center, University of Helsinki and Helsinki University Hospital, Helsinki, Finland; Meilahti Infectious Diseases and Vaccine Research Center, MeiVac, University of Helsinki and Helsinki University Hospital, Helsinki, Finland; Multidisciplinary Center of Excellence in Antimicrobial Resistance Research, University of Helsinki, Helsinki, Finland; Human Microbiome Research Program, Faculty of Medicine, University of Helsinki, Helsinki, Finland

**Keywords:** ESBL, hospitalization, MDR bacteria, MDRO, MRSA

## Abstract

**Background:**

As hospitals have a high prevalence of multidrug-resistant organisms (MDRO), hospitalization abroad indicates for travellers an increased risk of acquiring MDRO—and carrying the strains home. Antimicrobial resistance (AMR) rates are highest in the (sub)tropics, whereas Europe is considered a lower risk region. Since AMR prevalences vary within Europe, we aimed to gather country-specific data on the risks for hospitalized travellers.

**Methods:**

At hospitals of the Helsinki and Uusimaa district in Finland, patients hospitalized abroad over the past 12 months are systematically screened for methicillin-resistant *Staphylococcus aureus* (MRSA), extended-spectrum beta-lactamase (ESBL)-producing Enterobacterales (ESBL-PE), carbapenemase-producing bacteria and vancomycin-resistant *Enterococcus* spp. (VRE). Among patients screened 2010–19, we selected those hospitalized in Europe, recorded their MDRO findings, infections and mortality, and analysed MDRO-associated risk factors.

**Results:**

Of the 1772 patients treated in 41 European countries, 16.6% (295) carried MDRO, 12.5% (221) ESBL-PE, 7.8% (138) solely ESBL-*E. coli*, 2.6% (46) MRSA, 2.2% (30) of those screened VRE and 2.2% (39) carbapenem-resistant Gram-negatives. Among those colonized, 9.8% (29) had symptomatic MDRO infections and 0.3% (one) died. Colonization was most frequently recorded for those treated in eastern and southern Europe, with Bulgaria, Cyprus and the Russian Federation scoring highest. MDRO colonization was associated with antibiotic treatment and showed a negative correlation with time from discharge to screening.

**Conclusions:**

After hospitalization in European countries, ESBL-PE carriage was relatively common (12.5%), while other MDROs proved less frequent (<5%). Antibiotic treatment and short time since hospitalization abroad increased the risk of MDRO colonization. Clear differences between countries and regions were revealed, with highest rates in the east and the south.

## Introduction

A major accelerator of the increase in global antimicrobial resistance (AMR) is its spread from high- to lower prevalence regions through travel and trade.^1^ Consistent with the high prevalence of multidrug-resistant organisms (MDROs) in emerging economies, a multitude of studies show that 20–70% of visitors to these regions are colonized by MDRO on return home.^1^ AMR transfer between high-income countries has received scant attention.

Travellers hospitalized abroad are at particular risk of acquiring resistant bacteria. Indeed, several investigations in Europe report MDRO carriage rates as high as 44% after healthcare contact abroad.^2–9^ According with other traveller studies, the risk varies by region: in our previous investigation in Finland screening for MDRO among patients hospitalized abroad, the highest risk was associated with the Indian subcontinent followed by Southeast Asia, Africa and South America.^10^ Those treated in Europe had considerably lower carriage rates than those treated in (sub)tropical regions.^10^ However, considerable differences exist in AMR prevalence between the various European countries, as shown by laboratory data published by the European Antimicrobial Resistance Surveillance Network (EARS-Net), for example.^11^

Although our earlier investigation showed a smaller health risk for visits to western and eastern European destinations than to the (sub)tropics,^12^ detailed research into travels within Europe is warranted.^10^^,^^12^ We found very limited data, only three studies providing comparisons of healthcare-related risks of MDRO colonization by destination countries in Europe.^5^^,^^7^^,^^8^ Therefore, we undertook analysis of data on patients screened in Finland within a year after treatment and/or a major invasive procedure at hospitals in other European countries.

## Methods

### Study design and selection of participants

Drawing upon a regional infection control database run by the Helsinki and Uusimaa Hospital District with a population of 1.7 million, we identified patients screened for both methicillin-resistant *Staphylococcus aureus* (MRSA) and multidrug-resistant gram-negative bacteria (MDRGNB) within a 30-day time frame between January 2010 and August 2019. Our basic inclusion criteria comprised adequate records of screens involving (i) three-site MRSA cultures (nose, throat, and groin or perineum); and (ii) stool specimens or rectal swabs for MDRGNB cultures. Another criterion to be met in medical records was hospitalization for over 24 hours or a major invasive procedure while abroad in a European country within 12 months before screening. We defined as invasive any procedure which required anaesthesia or could not be carried out bedside, thus including for example any major surgery. A documented or deducible discharge date within a 30-day time frame was also required. Documented travel outside Europe or hospitalization in multiple countries over the preceding 12 months led to exclusion.

### Ethical statement

The research board of Helsinki University Hospital Department of Internal Medicine approved this study. In accordance with the Finnish Medical Research Act, an ethics committee review was not required, as there was no intervention.

### Collection of data on MDRO colonization and clinical infections

We recorded all MDROs found in screening and clinical samples within one month (31 days) of the first screening day. Our hospital district guidelines require that patients who have undergone 24-hour hospitalization or medical procedures abroad within 12 months should be systematically screened. This has applied to all countries since 2016, whereas the Nordic countries were excluded over 2010–16. During the study period, a minimum of three-site MRSA and a faecal/rectal MDRGNB screening on two separate days were advised. Over 2010–16, vancomycin-resistant *Enterococcus* (VRE) screening was instructed for all; since 2016 it has only been obligatory for direct transfer patients. The guidelines (including additional screening of catheter urine plus two sites, wound and throat/trachea), are shown in Supplementary Table 1. We also recorded microbiologically verified symptomatic MDRO infections and mortality associated to them during hospitalization in Finland (30 days maximum).

### Collection of data on risk factors

On the basis of medical records, the Charlson comorbidity index (CCI),^13^ verified or suspected alcohol abuse, surgery and intensive care unit (ICU) admission abroad were recorded. Antibiotic exposure abroad was listed for those with documented use, bacterial infection treatment or surgery routinely requiring prophylaxis; this included oral and parenteral antibiotics given before, during or after hospitalization prior to admission to our hospitals. These were all classified as negative, if not mentioned. Type of travel was recorded either as residence, known or suspected visit to friends and relatives (VFR), or work/holiday/other. The duration of hospitalization abroad was also listed, or in cases with multiple instances, the sum within 1 month.

For analysis of risk associated with European countries and subregions, three types of comparison were made: (i) individual countries, (ii) subregions (East, North, South, West) according to the United Nations classification except Cyprus which was classified as belonging to southern Europe^14^ and (iii) grouping by 2010–18 EARS-Net prevalence data, as shown in Supplementary Tables 2–3.^11^ The average rate of methicillin resistance among invasive *S. aureus* strains and, as a surrogate of ESBL-PE, third-generation cephalosporin resistance (3GCR) among invasive *E. coli* and *K. pneumoniae* strains were recorded.

**Figure 1:**
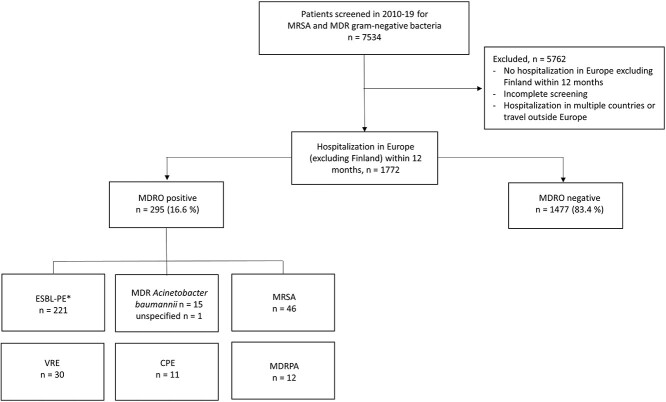
Identification of patients hospitalized in European countries and screened within 12 months in 2010–19 at the Helsinki University Hospital, Finland. *n* = number of patients. A single patient may have more than one strain of the same MDR bacterial class. *In addition, 10 patients carried non-ESBL Enterobacterales resistant to third-generation cephalosporins. Abbreviations: CPE = carbapenemase-producing Enterobacterales; ESBL-PE = extended-spectrum beta-lactamase-producing Enterobacterales; MDR = multidrug-resistant; MDRPA = multidrug-resistant *Pseudomonas aeruginosa*; MRSA = methicillin-resistant *Staphylococcus aureus*; VRE = vancomycin-resistant *Enterococcus*

### Microbiological methods

The microbiological methods in routine clinical use at the Helsinki University Hospital during the study were described in our earlier paper.^10^ In brief, MRSA detected after overnight enrichment by culture was confirmed using *S. aureus*-specific nuclease and *mecA* gene quantitative PCR.^10^ VRE was screened through enrichment by selective culture and confirmed by an in-house PCR.^10^ After culture on respective selective plates, the species of ESBL-PE and carbapenemase-producing Enterobacterales (CPE) were identified by matrix-assisted laser desorption ionization time-of-flight (MALDI-TOF; Vitek-MS, bioMérieux); for CPE, an in-house carbapenemase gene PCR was applied.^10^ Resistance was confirmed by the Clinical and Laboratory Standards Institute (CLSI)^15^ and, from 2011, the European Committee on Antimicrobial Susceptibility testing (EUCAST) methods.^16^

Multidrug-resistant *Acinetobacter* species (MDRACI) and *Pseudomonas aeruginosa* (MDRPA) isolates were retrieved from ESBL and KPC plates, and analysed further by C-390, VITEK-GN or MALDI-TOF. *Acinetobacter* isolates resistant to meropenem and *P. aeruginosa* isolates resistant to meropenem and ceftazidime were subjected to PCR analysis for carbapenemase genes.^10^

### Statistics

The statistical analyses were performed using SPSS v. 25.0 (IBM Corp., Armonk, NY, USA). Univariate analysis of risk factors was carried out by χ^2^ test, Fisher’s exact test or binary logistic regression. Independent variables with *P* values < 0.2 in univariate analysis were included in multivariable analysis if they did not correlate strongly. In multivariable logistic regression, backward selection based on Akaike information criteria was used.

## Results

### Patient characteristics

The final study population comprised 1772 patients (Figure 1) treated in 41 European countries, half (49.3%) of them in Spain or Estonia. Supplementary Table 4 presents patient characteristics for the seven countries with highest patient numbers and Tables 1–3 for the whole study population.

**Table 1:** Multidrug-resistant bacteria carriage and risk factor analysis of patients treated in other European countries within 12 months before screening in Finland 2010–19

	Number of patients	MDRO positive, *n* (%)	MDRO negative, *n* (%)	OR (95% CI), univariate analysis	*P* value, univariate analysis	AOR (95% CI, multivariable analysis)	*P* value, multivariable analysis
Total	1772	295 (16.6)	1477 (83.4)				
Sex							
Female	839	134 (16.0)	705 (84.0)	1.0	-		
Male	933	161 (17.3)	772 (82.7)	1.1 (0.9–1.4)	0.469		
Age group					0.834		
0–17	180	29 (16.1)	151 (83.9)	0.9 (0.6–1.4)	0.640		
18–30	186	26 (14.0)	160 (86.0)	0.8 (0.5–1.2)	0.246		
31–50	371	63 (17.0)	308 (83.0)	1.0 (0.7–1.3)	0.801		
51–65	416	68 (16.3)	348 (83.7)	0.9 (0.7–1.3)	0.597		
Over 65	619	109 (17.6)	510 (82.4)	1.0	–		
CCI					0.051	–^b^	–^b^
0–1	1155	180 (15.6)	975 (84.4)	1.0	–		
2–4	463	79 (17.1)	384 (82.9)	1.1 (0.8–1.5)	0.464		
Over 4	154	36 (23.4)	118 (76.6)	1.7 (1.1–2.5)	0.015		
Alcohol abuse						–^b^	–^b^
Yes	147	31 (21.1)	116 (78.9)	1.4 (0.9–2.1)	0.133		
No	1625	264 (16.2)	1361 (83.8)	1.0	-		
Travel type					0.211		
Work/holiday	1180	184 (15.6)	996 (84.4)	1.0	–		
Residence	370	67 (18.1)	303 (81.9)	1.2 (0.9–1.6)	0.252		
VFR	222	44 (19.8)	178 (80.2)	1.3 (0.9–1.9)	0.118		
Antibiotic use							
Yes	722	168 (23.3)	554 (76.7)	2.2 (1.7–2.8)	<0.001	1.9 (1.5–2.5)	<0.001
No	1050	127 (12.1)	923 (87.9)	1.0	–	1.0	–
ICU treatment							
Yes	245	60 (24.5)	185 (75.5)	1.8 (1.3–2.5)	<0.001	1.4 (1.0–2.0)	0.064
No	1527	235 (15.4)	1292 (84.6)	1.0	-	1.0	-
Invasive procedure							
Yes	710	135 (19.0)	575 (81.0)	1.3 (1.0–1.7)	0.029	1.3 (1.0–1.7)	0.068
No	1062	160 (15.1)	902 (84.9)	1.0	–	1.0	–
Time from discharge to screening; days, median (IQR)^c^	6 (53)	3 (26)	7 (58)	0.9 (0.8–1.0)	<0.001	0.9 (0.9–1.0)	0.002
Country^a^					<0.001		<0.001
Spain	507	81 (16.0)	426 (84.0)	1.0 (0.7–1.3)	0.819	0.9 (0.7–1.3)	0.742
Estonia	366	41 (11.2)	325 (88.8)	0.6 (0.4–0.9)	0.018	0.7 (0.5–1.0)	0.049
Russian fed.	110	29 (26.4)	81 (73.6)	1.8 (1.2–2.9)	0.009	2.1 (1.3–3.3)	0.002
Germany	108	13 (12.0)	95 (88.0)	0.7 (0.4–1.2)	0.220	0.7 (0.4–1.2)	0.174
Greece	100	19 (19.0)	81 (81.0)	1.2 (0.7–2.0)	0.507	1.1 (0.7–1.9)	0.614
Italy	74	12 (16.2)	62 (83.8)	1.0 (0.5–1.8)	0.954	1.0 (0.5–1.8)	0.925
France	70	11 (15.7)	59 (84.3)	0.9 (0.5–1.8)	0.865	0.9 (0.5–1.8)	0.823
UK	43	2 (4.7)	41 (95.3)	0.2 (0.1–1.0)	0.043	0.3 (0.1–1.0)	0.047
Austria	38	7 (18.4)	31 (81.6)	1.1 (0.5–2.5)	0.738	1.0 (0.4–2.2)	0.958
Portugal	35	6 (17.1)	29 (82.9)	1.1 (0.4–2.5)	0.911	1.2 (0.5–2.9)	0.665
Sweden	33	2 (6.1)	31 (93.9)	0.3 (0.1–1.3)	0.107	0.3 (0.1–1.1)	0.069
Poland	29	6 (20.7)	23 (79.3)	1.3 (0.6–3.2)	0.527	1.3 (0.5–3.2)	0.541
Latvia	23	3 (13.0)	20 (87.0)	0.8 (0.2–2.4)	0.644	0.8 (0.3–2.5)	0.652
Cyprus	22	7 (31.8)	15 (68.2)	2.4 (1.0–5.6)	0.052	2.3 (0.9–5.5)	0.067
Bulgaria	21	8 (38.1)	13 (61.9)	3.1 (1.3–7.3)	0.009	3.1 (1.3–7.4)	0.012
Switzerland	20	3 (15.0)	17 (85.0)	0.9 (0.3–2.9)	0.854	1.0 (0.3–3.2)	0.964
Other	173	45 (26.0)	128 (74.0)	1.8 (1.2–2.6)	0.003	1.9 (1.3–2.8)	0.002
Subregion					<0.001		
Eastern	201	57 (28.4)	144 (71.6)	3.3 (2.2–5.0)	<0.001	N/A	N/A
Northern	496	53 (10.7)	443 (89.3)	1.0	–	N/A	N/A
Southern	796	144 (18.1)	652 (81.9)	1.8 (1.3–2.6)	<0.001	N/A	N/A
Western	279	41 (14.7)	238 (85.3)	1.4 (0.9–2.2)	0.102	N/A	N/A

^a^Deviation from the overall level was determined for each country in univariate and multivariable analyses.

^b^Dropped out before final step in backward selection.

^c^Analysed as a continuous variable in univariate and multivariable analysis with OR and AOR per 30 days.

AOR = adjusted odds ratio; CCI = Charlson comorbidity index; CI = confidence interval; ICU = intensive care unit; IQR = interquartile range; MDRO = multidrug-resistant organism; N/A = not applicable; OR = odds ratio; VFR = visiting friends and relatives.

**Table 2:** Extended-spectrum beta-lactamase-producing Enterobacterales (ESBL-PE) carriage and risk factor analysis of patients treated in other European countries within 12 months before screening in Finland 2010–19

	Number of patients	ESBL-PE positive, *n* (%)	ESBL-PE negative, *n* (%)	OR (95% CI), univariate analysis	*P* value, univariate analysis	AOR (95% CI, multivariable analysis)	*P* value, multivariable analysis
Total	1772	221 (12.5)	1551 (87.5)				
Sex							
Female	839	102 (12.2)	737 (87.8)	1.0	-		
Male	933	119 (12.8)	814 (87.2)	1.1 (0.8–1.4)	0.704		
Age group					0.516		
0–17	180	25 (13.9)	155 (86.1)	1.3 (0.8–2.1)	0.286		
18–30	186	20 (10.8)	166 (89.2)	1.0 (0.6–1.7)	0.929		
31–50	371	52 (14.0)	319 (86.0)	1.3 (0.9–1.9)	0.158		
51–65	416	56 (13.5)	360 (86.5)	1.3 (0.9–1.8)	0.230		
Over 65	619	68 (11.0)	551 (89.0)	1.0	–		
CCI					0.604		
0–1	1155	143 (12.4)	1012 (87.6)	1.0	–		
2–4	463	55 (11.9)	408 (88.1)	1.0 (0.7–1.3)	0.781		
Over 4	154	23 (14.9)	131 (85.1)	1.2 (0.8–2.0)	0.372		
Alcohol abuse							
Yes	147	21 (14.3)	126 (85.7)	1.2 (0.7–1.9)	0.487		
No	1625	200 (12.3)	1425 (87.7)	1.0	–		
Travel type					0.440		
Work/holiday	1180	140 (11.9)	1040 (88.1)	1.0	–		
Residence	370	48 (13.0)	322 (87.0)	1.1 (0.8–1.6)	0.569		
VFR	222	33 (14.9)	189 (85.1)	1.3 (0.9–2.0)	0.213		
Antibiotic use							
Yes	722	112 (15.5)	610 (84.5)	1.6 (1.2–2.1)	0.001	1.5 (1.1–2.0)	0.012
No	1050	109 (10.4)	941 (89.6)	1.0	–	1.0	–
ICU treatment							
Yes	245	41 (16.7)	204 (83.3)	1.5 (1.0–2.2)	0.030	1.4 (0.9–2.1)	0.096
No	1527	180 (11.8)	1347 (88.2)	1.0	–	1.0	–
Invasive procedure							
Yes	710	97 (13.7)	613 (86.3)	1.2 (0.9–1.6)	0.215		
No	1062	124 (11.7)	938 (88.3)	1.0	–		
Time from discharge to screening; days, median (IQR)^c^	6 (53)	4 (28)	7 (57)	0.9 (0.9–1.0)	0.015	0.9 (0.9–1.0)	0.044
Country^a^					<0.001		<0.001
Spain	507	51 (10.1)	456 (89.9)	0.7 (0.5–1.1)	0.110	0.7 (0.5–1.0)	0.078
Estonia	366	32 (8.7)	334 (91.3)	0.6 (0.4–1.0)	0.034	0.7 (0.4–1.0)	0.064
Russian fed.	110	27 (24.5)	83 (75.5)	2.2 (1.4–3.5)	0.001	2.4 (1.5–3.8)	<0.001
Germany	108	9 (8.3)	99 (91.7)	0.6 (0.3–1.2)	0.151	0.6 (0.3–1.2)	0.142
Greece	100	13 (13.0)	87 (87.0)	1.0 (0.6–1.8)	0.992	1.0 (0.5–1.8)	0.904
Italy	74	8 (10.8)	66 (89.2)	0.8 (0.4–1.7)	0.568	0.8 (0.4–1.6)	0.513
France	70	8 (11.4)	62 (88.6)	0.9 (0.4–1.8)	0.688	0.9 (0.4–1.8)	0.699
UK	43	1 (2.3)	42 (97.7)	0.2 (0.0–1.0)	0.055	0.2 (0.0–1.1)	0.061
Austria	38	6 (15.8)	32 (84.2)	1.3 (0.5–2.9)	0.606	1.1 (0.5–2.7)	0.773
Portugal	35	3 (8.6)	32 (91.4)	0.6 (0.2–1.9)	0.418	0.7 (0.2–2.1)	0.485
Sweden	33	2 (6.1)	31 (93.9)	0.4 (0.1–1.7)	0.226	0.4 (0.1–1.6)	0.186
Poland	29	6 (20.7)	23 (79.3)	1.7 (0.7–4.2)	0.215	1.8 (0.7–4.3)	0.201
Latvia	23	3 (13.0)	20 (87.0)	1.0 (0.3–3.2)	0.999	1.0 (0.3–3.2)	0.992
Cyprus	22	7 (31.8)	15 (68.2)	3.1 (1.3–7.5)	0.011	3.0 (1.3–7.3)	0.014
Bulgaria	21	8 (38.1)	13 (61.9)	4.1 (1.7–9.7)	0.001	4.1 (1.7–9.7)	0.002
Switzerland	20	3 (15.0)	17 (85.0)	1.2 (0.4–3.8)	0.785	1.3 (0.4–4.1)	0.709
Other	173	34 (19.7)	139 (80.3)	1.6 (1.1–2.5)	0.023	1.6 (1.1–2.5)	0.022
Subregion					<0.001	N/A	N/A
Eastern	201	50 (24.9)	151 (75.1)	3.5 (2.2–5.5)	<0.001	N/A	N/A
Northern	496	43 (8.7)	453 (91.3)	1.0	–	N/A	N/A
Southern	796	96 (12.1)	700 (87.9)	1.4 (1.0–2.1)	0.057	N/A	N/A
Western	279	32 (11.5)	247 (88.5)	1.4 (0.8–2.2)	0.207	N/A	N/A
*E. coli* and *K. pneumoniae* 3GCR country prevalence^b^					0.003	N/A	N/A
<10%	108	13 (12.0)	95 (88.0)	0.7 (0.3–1.3)	0.227	N/A	N/A
10–25%	1205	118 (9.8)	1087 (90.2)	0.5 (0.4.-0.8)	<0.001	N/A	N/A
>25%	288	49 (17.0)	239 (83.0)	1.0	–	N/A	N/A

^a^Deviation from the overall level was determined for each country in univariate and multivariable analyses.

^b^Third-generation cephalosporin resistance prevalence in invasive (blood or cerebrospinal fluid) *E. coli* and *K. pneumoniae* isolates 2010–2018 reported by EARS-Net. Data missing for 171 patients.

^c^Analysed as a continuous variable in univariate and multivariable analysis with OR and AOR per 30 days.

3GCR = Third-generation cephalosporin resistance; AOR = adjusted odds ratio; CCI = Charlson comorbidity index; CI = confidence interval; ICU = intensive care unit; IQR = interquartile range; MDRO = multidrug-resistant organism; N/A = not applicable; OR = Odds ratio; VFR = visiting friends and relatives.

**Table 3:** Methicillin-resistant *Staphylococcus aureus* (MRSA) carriage and risk factor analysis of patients treated in other European countries within 12 months before screening in Finland 2010–19

	Number of patients	MRSA positive, *n* (%)	MRSA negative, *n* (%)	OR (95% CI), univariate analysis	*P* value, univariate analysis	AOR (95% CI, multivariable analysis)	*P* value, multivariable analysis
Total	1772	46 (2.6)	1726 (97.4)				
Sex							
Female	839	17 (2.0)	822 (98.0)	1.0	–	–^a^	–^a^
Male	933	29 (3.1)	904 (96.9)	1.6 (0.8–2.8)	0.156		
Age group					0.367		
0–17	180	5 (2.8)	175 (97.2)	0.9 (0.3–2.3)	0.759		
18–30	186	3 (1.6)	183 (98.4)	0.5 (0.1–1.7)	0.255		
31–50	371	12 (3.2)	359 (96.8)	1.0 (0.5–2.1)	0.998		
51–65	416	6 (1.4)	410 (98.6)	0.4 (0.2–1.1)	0.079		
Over 65	619	20 (3.2)	599 (96.8)	1.0	–		
CCI					0.272		
0–1	1155	29 (2.5)	1126 (97.5)	1.0	–		
2–4	463	10 (2.2)	453 (97.8)	0.9 (0.4–1.8)	0.678		
Over 4	154	7 (4.5)	147 (95.5)	1.8 (0.8–4.3)	0.153		
Alcohol abuse							
Yes	147	10 (6.8)	137 (93.2)	3.2 (1.6–6.6)	0.001	3.5 (1.7–7.4)	<0.001
No	1625	36 (2.2)	1589 (97.8)	1.0	–	1.0	–
Travel type					0.335		
Work/holiday	1180	26 (2.2)	1154 (97.8)	1.0	–		
Residence	370	13 (3.5)	357 (96.5)	1.6 (0.8–3.2)	0.164		
VFR	222	7 (3.2)	215 (96.8)	1.4 (0.6–3.4)	0.394		
Antibiotic use							
Yes	722	32 (4.4)	690 (95.6)	3.4 (1.8–6.5)	<0.001	3.1 (1.6–6.1)	<0.001
No	1050	14 (1.3)	1036 (98.7)	1.0	–	1.0	–
ICU treatment							
Yes	245	7 (2.9)	238 (97.1)	1.1 (0.5–2.5)	0.782		
No	1527	39 (2.6)	1488 (97.4)	1.0	-		
Invasive procedure							
Yes	710	24 (3.4)	686 (96.6)	1.7 (0.9–3.0)	0.093	–^a^	–^a^
No	1062	22 (2.1)	1040 (97.9)	1.0	–		
Time from discharge to screening; days, median (IQR)^b^	6 (53)	4 (54)	6 (53)	0.9 (0.8–1.1)	0.326		
Country^c^					0.955		
Spain	507	18 (3.6)	489 (96.4)	1.6 (0.7–3.8)	0.246		
Estonia	366	8 (2.2)	358 (97.8)	1.0	–		
Russian fed.	110	2 (1.8)	108 (98.2)	0.8 (0.2–4.0)	0.814		
Germany	108	2 (1.9)	106 (98.1)	0.8 (0.2–4.0)	0.832		
Greece	100	0 (0)	100 (100)	N/A	N/A		
Italy	74	1 (1.4)	73 (98.6)	0.6 (0.1–5.0)	0.647		
France	70	3 (4.3)	67 (95.7)	2.0 (0.5–7.7)	0.314		
UK	43	0 (0)	43 (100)	N/A	N/A		
Austria	38	0 (0)	38 (100)	N/A	N/A		
Portugal	35	3 (8.6)	32 (91.4)	4.2 (1.1–16.6)	0.041		
Sweden	33	0 (0)	33 (100)	N/A	N/A		
Poland	29	0 (0)	29 (100)	N/A	N/A		
Latvia	23	0 (0)	23 (100)	N/A	N/A		
Cyprus	22	0 (0)	22 (100)	N/A	N/A		
Bulgaria	21	1 (4.8)	20 (95.2)	2.2 (0.3–18.8)	0.458		
Switzerland	20	0 (0)	20 (100)	N/A	N/A		
Other	173	8 (4.6)	165 (95.4)	2.2 (0.8–5.9)	0.128		
Subregion					0.166	N/A	N/A
Eastern	201	5 (2.5)	196 (97.5)	1.6 (0.5–4.8)	0.443	N/A	N/A
Northern	496	8 (1.6)	488 (98.4)	1.0	–	N/A	N/A
Southern	796	28 (3.5)	768 (96.5)	2.2 (1.0–4.9)	0.048	N/A	N/A
Western	279	5 (1.8)	274 (98.2)	1.1 (0.4–3.4)	0.852	N/A	N/A
Country MRSA prevalence^d^					0.108	–^a^	–^a^
<10%	548	8 (1.5)	540 (98.5)	1.0	–		
10–25%	281	7 (2.5)	274 (97.5)	1.7 (0.6–4.8)	0.297		
>25%	772	26 (3.4)	746 (96.6)	2.4 (1.1–5.2)	0.036		

^a^Dropped out before final step in backward selection.

^b^Analysed as a continuous variable in univariate and multivariable analysis with OR and AOR per 30 days.

^c^Deviation from the overall level was determined for each country in univariate and multivariable analyses.

^d^Prevalence of methicillin resistance among invasive (blood and/or cerebrospinal fluid) *S. aureus* isolates 2010–18 reported by EARS-Net. Data missing for 171 patients.

AOR = Adjusted odds ratio; CCI = Charlson comorbidity Index; CI = confidence interval; ICU = intensive care unit; IQR = interquartile range; MRSA = Methicillin-resistant *S. aureus*; N/A = not applicable; OR = odds ratio; VFR = visiting friends and relatives.

### MDRO colonization and risk factors

**Figure 2:**
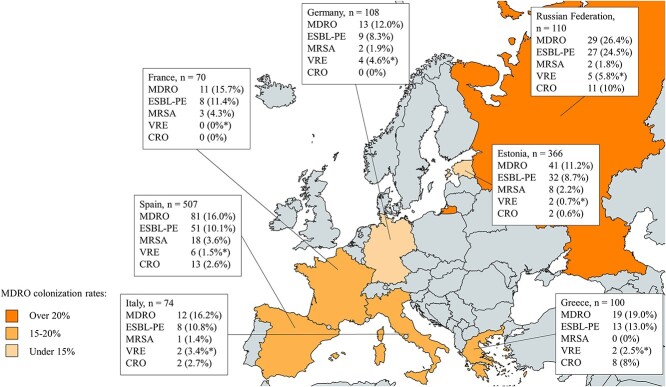
Carriage of multidrug-resistant organisms (MDROs) among patients treated at hospitals in other European countries within 12 months before screening in Finland. Data are shown for the seven countries with the highest patient tallies in Helsinki University Hospital records over 2010–19. Abbreviations: CRO = carbapenem-resistant organisms including carbapenemase-producing Enterobacterales, multidrug-resistant (MDR) *Acinetobacter* species and MDR *Pseudomonas aeruginosa*; ESBL-PE = extended-spectrum beta-lactamase-producing Enterobacterales; MRSA = methicillin-resistant *Staphylococcus aureus*; VRE = vancomycin-resistant *Enterococcus.* *Proportion of colonized individuals among those screened for VRE. Map created with mapchart.net ©

A total of 16.6% of the patients (295) carried MDRO. Among the countries with at least 20 patients, the highest MDRO rates were seen for Bulgaria (38.1%), Cyprus (31.8%) and the Russian Federation (26.4%) (Table 1). In comparisons between the four subregions, eastern Europe showed the greatest risk of colonization by MDRO and ESBL-PE. High country-specific prevalences of MRSA and 3GCR among *E. coli* and *K. pneumoniae* were associated with increased rates of MRSA and ESBL-PE colonization in our data, respectively. Figure 2 presents MDRO findings for the seven most common countries of hospitalization. The annual MDRO rates for the total study population over 2010–19 varied between 13.3% and 21.0%, the differences not reaching statistical significance (data not shown).

Of the various MDROs, ESBL-PE were carried by 12.5% of the patients (221), ESBL *K. pneumoniae* by 2.8% (49), MRSA by 2.6% (46), VRE by 2.2% (30 of 1394 individuals screened), MDRACI by 0.9% (16), MDRPA by 0.7% (12) and CPE by 0.6% (11). A total of 7.8% (138) of the entire study population were colonized only by ESBL *E. coli* strains. Thus, considering only MDROs other than ESBL *E. coli*, 8.9% (157) were colonized. In addition, 0.6% (10) had 3GCR (non-ESBL) Enterobacterales included neither in the total rate nor the analyses.

Fourteen patients showed carbapenemase gene-positive MDR *Acinetobacter baumannii;* two *Acinetobacter* strains had not been tested. Of the 12 individuals with MDRPA, three had carbapenemase gene-positive and five gene-negative strains; the isolates of four had not been tested.

The MDRO rates were higher among ICU-treated than non-ICU-treated patients: 24.5% (60/245) vs 15.4% (235/1527), odds ratio 1.8 (95% CI 1.3–2.5), *P* < 0.001. A detailed list of the MDRO findings is provided in Supplementary Table 5.

The risk factor analyses of MDRO, ESBL-PE and MRSA carriage are shown in Tables 1–3. Multivariable analysis revealed destination country and antibiotic use to be independently associated with MDRO carriage; increasing timespan from hospital discharge to screening showed a negative association. The same three associations were also found for ESBL-PE carriage. Antibiotic use and alcohol abuse were revealed as independent risk factors of acquiring MRSA.

To summarize the effects of the risk factors observed, we conducted a subgroup analysis of patients having been given antibiotics and screened within 30 days of discharge, vs no antibiotics and an interval of over 90 days, and found MDRO colonization rates of 26.1% (141/541) and 9.2% (20/217), respectively.

### Clinical infections caused by MDROs after return to Finland

Of the MDRO carriers, 9.8% (29) had a symptomatic, microbiologically verified MDRO infection during hospitalization in Finland, and 0.3% (1) died. The most common diagnoses were urinary tract infection (UTI) (10 patients, 3.4%), followed by infections of surgical sites (seven patients, 2.4%). Four patients had MDRO bacteraemia (1.4%).

## Discussion

Although for travellers the risk of acquiring MDRO is highest in the (sub)tropics,^1^^,^^10^ colonization proved common (16.6%) also among our 1772 cases recently treated in European hospitals. Our data reveal substantial variation in the risk by country and European subregion.

### MDRO colonization after hospitalization in various parts of Europe

The MDRO rates were highest in the east, followed by southern, western and northern subregions, in this order. Although geographic grouping of countries is not ideal due to inter-country differences, this general finding accords with other studies, such as the north-to-south and west-to-east AMR gradients reported by EARS-Net.^11^ Similar gradients have also been shown by others. Kaiser *et al.* explored gentamicin-resistant gram-negative bacteria among hospital patients repatriated to the Netherlands in 1998–2001. Among those treated in Europe the highest rates were found for patients returning from the east and south.^8^ An ICU survey study by Lepape *et al.* concluded that AMR is more frequent in eastern and southern regions than elsewhere in Europe.^17^ Likewise, analysing resistance genes in wastewaters of seven European countries, Pärnänen *et al.* found a north–south AMR gradient.^18^

As logically expected, we found an association between high MRSA background prevalence and higher MRSA carriage rates. Similarly, patients treated in countries with a high *E. coli* and *K. pneumoniae* 3GCR prevalence showed the greatest rates of ESBL-PE colonization.

### Carriage of various MDRO types

ESBL-*E. coli* was clearly the most common finding, whereas carbapenem-resistant organisms (CPE, MDRACI and MDRPA) proved rare: phenotypic analyses showed that only 2.2% (39) of the patients carried one or more such strains. Although the MRSA colonization rates, 2.6% overall, proved quite low, they exceed the prevalence in Finland.^19^ Our finding agrees with previous studies reporting MRSA rates of 2.7% and 2.4% among patients treated in European countries.^8^^,^^10^ The differences in MRSA and ESBL-PE rates presumably relate to their modes of transmission: ESBL-PE is mainly contracted through food and contact while MRSA is acquired by direct contact.

It should be noted that 8.9% (157) of the study population were colonized by MDROs other than ESBL *E. coli*. The possibility of their spread within healthcare warrants a systematic screening strategy.

### Clinical MDRO infections

A total of 9.8% of colonized individuals had symptomatic MDRO infections, consistent with the rate we earlier reported for patients hospitalized abroad around the world.^10^ We lacked data on infections treated during travel and those detected after hospital discharge in Finland, thus the actual rate may have been higher, but clearly MDRO carriers are asymptomatic for the most part. The development of symptoms depends on patient-related factors, but also on the bacterial strain. For example, ESBL-PE strains can be further characterized by analysing their virulence factors, as in the case of ESBL-producing diarrhoeagenic (ESBL-DEC), extraintestinal pathogenic (ESBL-ExPEC) and uropathogenic *E. coli* (ESBL-UPEC).^20^

### Risk factor analyses

In addition to risks associated with specific countries and subregions, antibiotic use and a short time span since hospitalization abroad were identified as factors independently associated with colonization by MDROs and ESBL-PE. Although in univariable analysis associated with an increased MDRO risk, ICU treatment and invasive procedures remained below statistical significance in multivariable analysis. As in previous studies, antibiotic use proved an independent risk factor for MDRO, ESBL-PE and MRSA carriage,^10^^,^^21–27^ pointing to the harmful effect the drugs have on the microbiota, thus facilitating MDRO acquisition.^21^ Indeed, if a patient reports taking any antibiotics during recent travel, that indicates an elevated risk of colonization upon return.^10^^,^^21–23^

Colonization dynamics easily explain the association between long time from hospital discharge and reduced risk of carriage: travel-acquired ESBL-PE colonization is often transient, i.e. these bacteria gradually disappear after return to low prevalence regions.^22^^,^^23^^,^^28^^,^^29^ We also reported the same association among travellers hospitalized in the (sub)tropics.^10^ Similarly, other MDROs can be cleared over time, as shown in studies among hospital patients and residents of long-term care facilities.^30^^,^^31^ We did not find any association between MRSA and time from discharge, consistent with the longer duration of colonization by MRSA than by ESBL-PE.^22^^,^^23^^,^^28^^,^^29^^,^^31^

Finally, our data could support hospital infection control by elucidating MDRO colonization risk related to hospitalization in various European countries and other risk factors. For example, there was a marked difference (26.1% vs 9.2%) between those having used antibiotics during travel who were screened within 30 days and those not having taken antibiotics who were screened only after 90 days.

### Limitations

Due to the retrospective design, we only drew on data available in medical records. It is plausible that not all patients were specifically asked about hospitalization abroad and, therefore, we may have missed some screenings. However, a severe illness or trauma leading to hospitalization would presumably be more likely to be reported even spontaneously, than minor visits to hospital, which were not in the focus of this study.

We could, of course, not rule out the possibility of the MDROs being acquired outside healthcare at the destination, particularly ESBL *E. coli* also spreading commonly in community settings.^32^ ESBL-PE may be contracted outside hospitals also in Europe: compiling the figures from a review by Armand-Lefèvre *et al.*, a colonization rate of 7 out of 120 (5.8%) is seen for visitors to Europe.^33^ This rate may be overestimated though since pre-travel colonization was not ruled out in all studies and some only included visitors to parts of Europe with highest background prevalence. We believe that part of the ESBL-PE in the present study may have been acquired outside hospitals yet the hospitalization increases the rates substantially. Indeed, a recent systematic review estimated that, overall, MDR colonization risk is doubled among travellers with healthcare contact.^34^

For some patients, colonization could have taken place already before travel or they may have contracted the bacteria after return to Finland before screening. However, because of the low MDRO background prevalence in Finland we believe these cases only constituted a minority^19^: our ESBL-PE rates of 12.5% substantially exceed the pre-travel ESBL-PE rate of 1.2% recorded for 430 Finnish travellers 2009–10,^21^ the rates of 4.7% for ESBL- *E. coli* and 1.1% for ESBL-*K. pneumoniae* recorded for medical students and elective surgery patients 2015–17,^35^ and the pre-travel rates of 4.4% for 750 Finnish travellers over 2017–19 (Kantele, unpublished observation).

## Conclusions

MDRO colonization, especially by ESBL-PE, proved relatively common among travellers who had been hospitalized and/or undergone major invasive procedures in Europe, yet differences were observed between the various subregions and countries, the highest risk associated with the east and the south. Increased MDRO rates correlated with antibiotic use and short time from discharge abroad. One in every ten colonized patients had a clinical MDRO infection. Screening at hospitals should also cover those hospitalized in Europe.

## List of abbreviations

AMR, antimicrobial resistance; AOR, adjusted odds ratio; CCI, Charlson comorbidity index; CI, confidence interval; CLSI, Clinical and Laboratory Standards Institute; CPE, carbapenemase-producing Enterobacterales; EARS-Net, European Antimicrobial Resistance Surveillance Network; *E. coli*, *Escherichia coli*; ESBL, extended-spectrum beta-lactamase; ESBL-PE, ESBL-producing Enterobacterales; ESBL-DEC, ESBL-producing diarrhoeagenic *E. coli*; ESBL-ExPEC, ESBL-producing extraintestinal pathogenic *E. coli*; ESBL-UPEC, ESBL-producing uropathogenic *E. coli*; EUCAST, European Committee on Antimicrobial Susceptibility testing; ICU, intensive care unit; IQR, interquartile range; KPC, *Klebsiella pneumoniae* carbapenemase; *K. pneumoniae*, *Klebsiella pneumoniae*; MALDI-TOF, matrix-assisted laser desorption ionization time-of-flight; MDR, multidrug-resistant; MDRACI, MDR *Acinetobacter* species; MDRGNB, MDR Gram-negative bacteria; MDRPA, MDR *Pseudomonas aeruginosa*; MDRO, multidrug-resistant organism; MRSA, methicillin-resistant *Staphylococcus aureus*; N/A, not applicable; OR, odds ratio; UTI, urinary tract infection; VFR, visit to friends and relatives; VRE, vancomycin-resistant *Enterococcus*; 3GCR, third-generation cephalosporin resistance

## Authors’ contributions

Study design was given by MK and AK; Data collection was done by MK and TK; Statistics were performed by MK; Drafting of manuscript was done by MK and AK; Critical comments on manuscript were given by TK; All authors had approved the final manuscript.

## Conflict of interest

MK has participated in a conference on the expense of Astellas Pharma. AK has received investigator-initiated grants (Valneva, Pfizer) and on an individual occasion consulted an advisory board (Valneva). None of the interests listed above are relevant to the current manuscript. TK reports no potential conflicts of interest.

## Supplementary Material

Kajova_JTM_MDRO_European_hospitals_supplementary_taac022
