# Supplementary material for: European hospitals as source of multidrug-resistant bacteria: analysis of travellers screened in Finland after hospitalization abroad
Source: J Travel Med. 2022 Mar 2;29(4):taac022. doi: 10.1093/jtm/taac022 (PMC9282090; doi:10.1093/jtm/taac022)
Supplement: Kajova_JTM_MDRO_European_hospitals_supplementary_taac022 [file kajova_jtm_mdro_european_hospitals_supplementary_taac022.pdf]

Supplementary Table 1. Summary of Helsinki University Hospital guidelines for multidrug-resistant (MDR) bacteria screening of patients hospitalized abroad.[a]

Issue year	Foreign countries covered	Number and timing of samples	Sampling sites
2010	All except the Nordic countries	Discharge from hospital abroad within 2 weeks: Sampling 3 times (0, 7 and 14 days from admission)<br><br>2 weeks – 12 months after discharge abroad: MRSA samples upon arrival; MDRGNB and VRE samples on 2 separate days.	MRSA: nose, throat, groin/perineum; if needed: wounds, catheter insertion sites, urine from indwelling catheter<br>MDRGNB: stool/rectum and throat/trachea[b]; if needed: wounds, urine for culture from indwelling catheter<br>VRE: stools; if needed: wounds
2012	All except the Nordic countries	Direct hospital transfer from abroad:<br>2 or 3 samples (MRSA and faecal MDRGNB: on days 0, 7 and 8; others: on days 0 and 7)<br><br>Hospitalization abroad within 12 months:<br>On separate days MRSA 1-2 times; MDRGNB and VRE 2 times	MRSA: nose, throat, groin/perineum; if needed: wounds, catheter/drain insertion sites, urine from indwelling catheter (if in place for over 7 days)<br>MDRGNB: stool/rectum; if needed: trachea[b], wounds, urine for culture from indwelling catheter (if in place for over 7 days)<br>VRE: stools/rectum; if needed: wounds
2016	All	All samples taken twice	MRSA: nose, throat, rectum; if needed: wounds, trachea[b],<br>urine from indwelling catheter (if in place for over 7 days)<br>MDRGNB: rectum; if needed: wounds, trachea[b],<br>urine from indwelling catheter (if in place for over 7 days)<br>VRE only in direct transfers from abroad: rectum; if needed: wounds, trachea[b], urine from indwelling catheter (if in place for over 7 days)
2019[c]	All	All samples taken twice	MRSA: nose, throat, rectum/perineum;<br>MDRGNB: rectum; if needed: wounds, trachea[b],<br>urine from indwelling catheter (if in place for over 7 days)<br>VRE only in direct transfers from abroad: rectum

Abbreviations: MDRGNB – multidrug-resistant Gram-negative bacteria screening package; MRSA – methicillin-resistant *Staphylococcus aureus*; VRE– vancomycin-resistant *Enterococcus* species

[a] Screening is recommended in cases of hospitalization abroad lasting over 24 hours and/or if a medical procedure has been performed. The table summarizes the Helsinki University Hospital guidelines translated by the authors.

[b] Applies to intubated/tracheostomized patients only.

Supplementary Table 2. Third-generation cephalosporin resistance of *Escherichia coli* and *Klebsiella pneumoniae*: prevalence categories for European countries used in extended-spectrum beta-lactamase-producing Enterobacterales (ESBL-PE) risk factor analyses.[a,b]

<10%<br>Total n = 108<br>(n)	10-25%<br>Total n = 1205<br>(n)	>25%<br>Total n = 288<br>(n)
Austria (38)	Belgium (18)	Bulgaria (21)
Denmark (4)	Croatia (15)	Czech Republic (9)
Iceland (1)	Estonia (366)	Cyprus (22)
Netherlands (18)	France (70)	Greece (100)
Norway (14)	Germany (108)	Italy (74)
Sweden (33)	Hungary (13)	Latvia (23)
	Ireland (4)	Poland (29)
	Lithuania (8)	Romania (8)
	Luxembourg (6)	Slovakia (2)
	Malta (9)	
	Portugal (35)	
	Slovenia (3)	
	Spain (507)	
	UK (43)	

[a] Prevalence of third-generation cephalosporin resistance in invasive (blood and cerebrospinal fluid) *Escherichia coli* and *Klebsiella pneumoniae* isolates 2010–18. Based on data published by the European Antimicrobial Resistance Surveillance Network (EARS-Net). Missing data for Croatia 2010, Poland 2014, and Slovakia 2010.

[b] Data missing (n = 171): Albania, Andorra, Bosnia and Herzegovina, Kosovo*, Monaco, Montenegro, North Macedonia, Republic of Moldova, Russian Federation, Serbia, Switzerland, Ukraine

*This designation is without prejudice to positions on status and accords with the United Nations Security Council Resolution 1244/99 and the International Court of Justice Opinion on the Kosovo declaration of independence.

Supplementary Table 3. Methicillin-resistant *Staphylococcus aureus* (MRSA) prevalence categories for European countries used in risk factor analyses.[a,b]

<10%<br>Total n = 548<br>(n)	10-25%<br>Total n = 281<br>(n)	>25%<br>Total n = 772<br>(n)
Austria (38)	Belgium (18)	Croatia (15)
Denmark (4)	Bulgaria (21)	Cyprus (22)
Estonia (366)	Czech Republic (9)	Greece (100)
Iceland (1)	France (70)	Italy (74)
Latvia (23)	Germany (108)	Malta (9)
Lithuania (8)	Hungary (13)	Portugal (35)
Netherlands (18)	Ireland (4)	Romania (8)
Norway (14)	Luxembourg (6)	Slovakia (2)
Sweden (33)	Poland (29)	Spain (507)
UK (43)	Slovenia (3)	

[a] MRSA prevalence in invasive (blood and/or cerebrospinal fluid) *Staphylococcus aureus* isolates. Based on data covering 2010–18 published by the European Antimicrobial Resistance Surveillance Network (EARS-Net). Data missing for Slovakia 2010.

[b] Data missing (n = 171): Albania, Andorra, Bosnia and Herzegovina, Kosovo*, Monaco, Montenegro, North Macedonia, Republic of Moldova, Russian Federation, Serbia, Switzerland, Ukraine

*This designation is without prejudice to positions on status and accords with the United Nations Security Council Resolution 1244/99 and the International Court of Justice Opinion on the Kosovo declaration of independence.

Supplementary Table 4. Characteristics of patients treated at hospitals in European countries within 12 months before screening for multidrug-resistant organisms (MDROs) in Finland. Data are only provided for the seven countries with the highest patient numbers in Helsinki University Hospital records over 2010–19.

	Spain	Estonia	Russian Federation	Germany	Greece	Italy	France
Number of patients	507	366	110	108	100	74	70
Sex							
Male, n (%)	300 (59.2)	178 (48.6)	55 (50.0)	48 (44.4)	48 (48.0)	39 (52.7)	37 (52.9)
Female, n (%)	207 (40.8)	188 (51.4)	55 (50.0)	60 (55.6)	52 (52.0)	35 (47.3)	33 (47.1)
Age median (IQR)	68 (16)	49.5 (37)	47 (31)	40.5 (33)	53.5 (35)	58.5 (29)	52.5 (35)
CCI median (IQR)	1 (3)	0 (2)	0 (2)	0 (1)	0 (2)	1 (2)	0 (2)
Alcohol abuse, n (%)	65 (12.8)	30 (8.2)	4 (3.6)	7 (6.5)	8 (8.0)	1 (1.4)	3 (4.3)
Travel type, n (%)							
Work/holiday/other	381 (75.1)	208 (56.8)	29 (26.4)	87 (80.6)	86 (86.0)	62 (83.8)	52 (74.3)
Residence	121 (23.9)	79 (21.6)	28 (25.5)	14 (13.0)	9 (9.0)	9 (12.2)	14 (20.0)
VFR	5 (1.0)	79 (21.6)	53 (48.2)	7 (6.5)	5 (5.0)	3 (4.1)	4 (5.7)
Surgery / invasive procedure, n (%)	165 (32.5)	175 (47.8)	44 (40.0)	61 (56.5)	38 (38.0)	19 (25.7)	38 (54.3)
ICU treatment, n (%)	87 (17.2)	47 (12.8)	8 (7.3)	20 (18.5)	12 (12.0)	8 (10.8)	9 (12.9)
Antibiotic use, n (%)	232 (45.8)	123 (33.6)	39 (35.5)	39 (36.1)	47 (47.0)	32 (43.2)	26 (37.1)
Days from discharge to screening, median (IQR)	8 (56)	8.5 (70)	10 (77)	12.5 (49)	1 (14)	2.5 (27)	4.5 (19)
Direct transfer, n (%)	170 (33.5)	134 (36.6)	37 (33.6)	33 (30.6)	44 (44.0)	30 (40.5)	27 (38.6)
Median duration of hospitalization abroad, days (IQR)	8 (10)	4 (6)	5 (10)	6 (13)	7 (7)	8 (8)	9 (10)
Duration of hosp. data missing, n (%)	164 (32.3)	142 (38.8)	37 (33.6)	35 (32.4)	21 (21.0)	21 (28.4)	23 (32.9)

CCI = Charlson comorbidity index; ICU = intensive care unit; IQR = interquartile range; VFR = visiting friends and relatives

Supplementary Table 5. Comparison of multidrug-resistant organism (MDRO) carriage among patients treated in intensive care units (ICU) in other European countries versus non-ICU-treated patients. The patients were screened at the Helsinki University Hospital 2010–19 within 90 days after discharge abroad.[a]

	ICU treatment (n = 213), n (%)	No ICU treatment (n = 1244), n (%)	OR (95% CI)	p value
Any MDROs	53 (24.9)	209 (16.8)	1.6 (1.2-2.3)	0.005
MDROs other than ESBL- *E. coli*	37 (17.4)	108 (8.7)	2.2 (1.5-3.3)	<0.001
ESBL-PE	34 (16.0)	160 (12.9)	1.3 (0.9-1.9)	0.219
ESBL- *K. pneumoniae*	11 (5.2)	35 (2.8)	1.9 (1.0-3.8)	0.074
VRE	10 (5.2)	20 (2.0)	2.7 (1.3-6.0)	0.011
MDRACI	9 (4.2)	7 (0.6)	7.8 (2.9-21.2)	<0.001
MRSA	7 (3.3)	31 (2.5)	1.3 (0.6-3.1)	0.503
MDRPA	6 (2.8)	5 (0.4)	7.2 (2.2-23.8)	0.001
CPE	5 (2.3)	6 (0.5)	5.0 (1.5-16.4)	0.009

CI = confidence interval; CPE = carbapenemase-producing Enterobacterales; ESBL-EC = extended-spectrum beta-lactamase-producing *Escherichia coli;* ESBL-KP = extended-spectrum beta-lactamase-producing *Klebsiella pneumoniae*; ESBL-PE = extended-spectrum beta-lactamase-producing Enterobacterales; ICU = intensive care unit; MDRACI = multidrug-resistant *Acinetobacter* species; MDRO = multidrug-resistant organisms; MDRPA = multidrug-resistant *Pseudomonas aeruginosa*; MRSA = methicillin-resistant *Staphylococcus aureus*; OR = odds ratio; VRE = vancomycin-resistant *Enterococcus* species

[a] Only patients screened within 90 days after hospital discharge abroad are included to avoid missing data on whether or not a patient was treated in an ICU.
